# Evaluation of Alkaline-Labile Sulfane Sulfur in Biological Samples: The Influence of Hyperhomocysteinemia and Oxidative Stress

**DOI:** 10.3390/biom16060784

**Published:** 2026-05-27

**Authors:** Alexander Vladimirovich Ivanov, Valery Vasil’evich Aleksandrin, Alexandra Vladimirova Mishina, Mikhail Aleksandrovich Popov, Ruslan Andreevich Maslennikov, Polina Alexandrovna Pudova, Alexander Gennadievich Filippov, Ekaterina Andreevna Sergeeva, Alisa Anatolyevna Sokolovskaya, Aleksey Aleksandrovich Moskovtsev, Maria Petrovna Kruglova, Victor Aleksandrovich Stupin, Ekaterina Vladimirovna Silina, Aslan Amirkhanovich Kubatiev

**Affiliations:** 1 Institute of General Pathology and Pathophysiology, Baltiyskaya St., 8, 125315 Moscow, Russia; aleksandrin-54@mail.ru (V.V.A.); aleksandramishin@gmail.com (A.V.M.); popovcardio88@mail.ru (M.A.P.); polinaklever28@gmail.com (P.A.P.); algf@yandex.ru (A.G.F.); katya96korn@mail.ru (E.A.S.); alice.sokolovskaya@gmail.com (A.A.S.); bioinf@mail.ru (A.A.M.); niiopp@mail.ru (A.A.K.); 2 Moscow Regional Research and Clinical Institute n.a. M.F. Vladimirskiy, Shchepkin St., 61/2, 129110 Moscow, Russia; rusmaslennikov@mail.ru; 3 Department of Pathological Physiology, I.M. Sechenov First Moscow State Medical University (Sechenov University), Trubetskaya St., 8, 119991 Moscow, Russia; marykruglova@live.ru (M.P.K.); silinaekaterina@mail.ru (E.V.S.); 4 Department of Hospital Surgery No. 1, Pirogov Russian National Research Medical University, Ostrovityanova St., 1, 117997 Moscow, Russia; stvictor@bk.ru

**Keywords:** biomarkers, hyperhomocysteinemia, oxidative stress, polysulfides, sulfane sulfur

## Abstract

Polysulfides play an important role in regulating many physiological processes, making their study relevant in health and disease. This study aims to improve their evaluation method by determining alkaline-labile sulfane sulfur (ALSS) and to test it on various biological samples, as well as on models of hyperhomocysteinemia and oxidative/reductive stress. The ALSS assay is based on the photometric determination of H_2_S, which is generated from polysulfides under alkaline conditions. The limit of quantitative detection was 3.3 nmol, interday reproducibility was 5.7% (blood plasma), and the yields of H_2_S precipitation and product extraction were 97.8 and 89.7%, respectively. The specificity of the approach, determined with two different methods of sulfide destruction, was 80% on a model mixture of bovine albumin or blood plasma. A small but noticeable matrix effect was revealed. ALSS of control rats (*n* = 15) and human plasma (*n* = 10) was 133 (128; 135) and 177.6 (171.8; 183.6) μmol/g protein, respectively. Decreased ALSS levels were observed in rats with mild hyperhomocysteinemia (*n* = 14) and in patients with acute stroke (*n* = 20). Under 24h oxidative stress, ALSS decreased from 14.8 ± 1.3 to 10.4 ± 1.0 μmol/g protein (*p* = 0.0055) in the THP-1 culture, while 96h reductive stress caused it to increase from 10.9 ± 0.9 to 15.9 ± 2.0 μmol/g protein, *p* = 0.00034. The method presented in this paper allows for the assessment of ALSS in various biological matrices and has demonstrated sensitivity to oxidative stress in vitro and hyperhomocysteinemia in vivo. This makes it a promising indicator of sulfur metabolism disorders in pathological conditions.

## 1. Introduction

H_2_S is a well-known gasotransmitter, along with CO and NO. Although known as a chemical substance for centuries, its role as an endogenous regulator of physiological processes has only been studied for a few decades [1]. During this time, a large number of its molecular targets have been identified, primarily enzymes whose H_2_S activity significantly and directly influences health and disease, for example, through heme binding or S-sulfhydration mechanisms. A large number of such targets determine the pleiotropic effects of H_2_S. Thus, the role of endogenous H_2_S in the regulation of cellular respiration was revealed [2], through mechanisms that prevent the development of pathological conditions such as oxidative stress, endoplasmic reticulum stress, NO metabolism disorder [3], endothelial dysfunction, atherosclerosis, and arterial hypertension, as well as mechanisms that promote angiogenesis [4,5]. The mechanisms of the protective and cytotoxic effects of H_2_S in the central nervous system are actively being studied [6,7,8,9,10]. Clinical observations reveal an association of low H_2_S levels with a wide range of cardiovascular diseases (arterial hypertension, coronary heart disease, heart failure, and myocardial infarction), diabetes mellitus, chronic kidney disease, and neurodegenerative diseases (Parkinson’s disease and Alzheimer’s disease) [11,12,13].

Approaches to the study of H_2_S metabolism can be divided into two categories: methods for determining H_2_S-generating activity (enzymatic and non-enzymatic) and methods for determining the content of various H_2_S pools in fluids, cells, and tissues. H_2_S readily passes through biological membranes under physiological conditions and reacts to form reactive sulfur species (RSS). Therefore, when discussing the biological effects of H_2_S, we usually mean its various RSS forms, including both very short-lived compounds, such as radicals (RS∙), and relatively stable ones (persulfides, polysulfides, and sulfur associated with prosthetic groups of enzymes), as well as products of the interaction of RSS with active forms of nitrogen. Compounds with the general formula RS_n_H and RSS_n_R (n ≥ 2) are also called supersulfides [14]. There are free (native), acid-labile (mainly sulfur from Fe-S clusters), reducible (or dithitreitol (DTT)-labile: hydropersulfides RSSH and hydropolyhydrosulfides RS_n_H), and alkaline-labile H_2_S (or sulfide) pools [15,16], as well as total reactive sulfide species (native + RSSH + RS_n_H) [17]. Together with other low-molecular-weight thiols and cysteine residues of proteins, H_2_S forms a single system that is in dynamic equilibrium [14]. Although supersulfides are present in biological matrices in appreciable amounts, their physiological relevance remains uncertain, especially with regard to protein-bound polysulfides [18].

For biomarker studies, relatively stable forms of RSS are typically of interest, as they can be captured during sample preparation and stored until analysis. The half-life of free H_2_S is on the order of seconds to minutes in biological matrices [19], making it difficult to determine its true levels. However, free H_2_S is the most frequently reported in clinical studies, and its content varies widely according to different data. For example, in some analytical studies, this pool was not detected at all [20,21], while according to other data, it constitutes submicromolar levels in blood plasma [22,23]. Levels of acid-labile and reducible H_2_S are significantly higher (several μM) [23]. A recently developed approach for determining total reactive sulfide species showed levels of ~15–20 μM in blood plasma [17]. In general, in clinical studies, depending on sample preparation and the method of determination, H_2_S levels vary from ~0.3 to 150 μM [11]. Regarding cell culture analysis, the variety of approaches to their analysis is well presented in the review [16].

A fairly significant proportion of low-molecular-weight aminothiols (cysteine—Cys; glutathione—GSH) were found in cells and tissues, which can be found in the form of persulfides, as well as (hydro)polysulfides [18,24]. This indicates the possibility of the presence of sulfane sulfur in proteins, which does not belong to the prosthetic groups of enzymes but is covalently linked to cysteine residues. Quite a few approaches have been developed for the determination of sulfane sulfur, mostly based on the reduction in hydropolysulfides, their derivatization, the separation of reaction products with GC, HPLC with fluorescence or MS detection, gel electrophoresis, and fluorescent probes [25]. However, unlike persulfides and hydropolysulfides, polysulfides (RS_n_R) are not sensitive to reducing agents such as DTT [26] and cannot be directly derivatized using thiol-specific agents.

It was previously found that the incubation of blood or albumin in strongly alkaline conditions resulted in the slow release of significant amounts of H_2_S, but the authors considered this to be an artifact of the desulfurization of SH-groups of proteins and amino acids [27]. An important work in the biochemistry literature of H_2_S fractions is that of Ikeda et al. [26], in which the authors identified a high content of sulfane sulfur in biological matrices (albumin, blood plasma, etc.), which originated from polysulfides, and this pool was not available for analysis using previously developed approaches. In particular, when using the traditional method of Wood’s cold cyanolysis, the level of sulfane sulfur in blood plasma was about 0.1 mM [28,29]; however, when using the Ikeda approach, this level was ~8 mM. Their method, called EMSP-MB (Elimination Method of Sulfide from Polysulfide followed with methylene blue (MB) sulfide detection assay) is based on the formation of H_2_S from RS_n_R under alkaline conditions in the presence of ascorbate, followed by the release of H_2_S as ZnS and synthesis of MB from H_2_S with photometric detection (660–670 nm) [30]. Using EMSP-MB, they found that in proteins with high Cys content, such as albumins, ~12–16 H_2_S molecules are released per protein molecule, while in blood plasma, they amount to levels of about 8 mM, which is several orders of magnitude higher than that of other RSS or aminothiol pools [26]. In other biological fluids (seminal fluid, saliva, and tears), sulfide levels were also found to be quite high (~100–1000 μM) [26,31]. Combined with the ease of determination, this makes the proposed approach very promising in clinical and experimental medicine. However, to our knowledge, it has only been used in two clinical studies to date [32,33].

Despite the simplicity and persuasiveness of EMSP-MB, a number of questions about this approach remain unanswered. For example, the authors hypothesized that ascorbic acid acts as a reducing agent, converting the sulfur in polysulfides into H_2_S [26]. However, its actual role and the necessity of its use remain questionable. The reaction conditions for the formation of H_2_S and MB have not been fully studied or optimized. Furthermore, MB synthesis is accompanied by the formation of byproducts that can interfere with the analysis. A number of the authors’ results did not correspond to the theoretical principles of the method, and the results of the analysis of model albumin mixtures conflicted with those of the blood plasma analysis. Thus, this work aims to improve the method for the evaluation of alkaline-labile sulfane sulfur (ALSS) in various biological matrices (blood, blood plasma, internal organs, and cell cultures).

## 2. Materials and Methods

### 2.1. Equipment

A capillary electrophoresis (CE) system Kapel-205 (Lumex, Saint Petersburg, Russia) was used with an unbound silica capillary of 50 μm i.d. and 38 cm total (31.5 cm effective) length. The absorption signal at 292 nm was registered at a frequency of 10 s^−1^. The temperature of the capillary was 25 °C. For pH measurements, a pH meter Ecotest-2000 pH meter (Econix, Moscow, Russia) was employed. Photometry was performed using a Lambda EZ 201 UV/Vis spectrometer (PerkinElmer, Shelton, CT, USA) at 663 nm using plastic cuvettes with an optical path length of 1 cm. A Conentrator Plus (Eppendorf AG, Hamburg, Germany) was used to dry the samples.

### 2.2. Chemicals

The chemicals used in this study include the following: acetonitrile (ACN) 99.9% (Komponent-Reactiv, Moscow, Russia); ascorbic acid 99–100.5%, Na_3_PO_4_·12H_2_O, and NaH_2_PO_4_·2H_2_O, Na_2_HPO_4_·2H_2_O (Panreac, Barcelona, Spain); ethanol 95% (RFK, Moscow, Russia); HCl 37% (Acros Organics); ammonium polysulfide solution and MB analytical-grade (Himmed, Moscow, Russia); NaCl >99.5% purum p.a. (Fluka AG, Buchs, Switzerland); NaOH (Dia-M, Moscow, Russia); bovine serum albumin (BSA) 96%, phosphoric acid 85%, CaCl_2_, FeCl_3_, Na_2_SO_3_, and Na_2_S_2_O_3_ (Rushim, Moscow, Russia); dodecyl sulfate Na or (SDS) (SERVA Electrophoresis GmbH, Heidelberg, Germany); L-Cys, cysteinylglycine, cystine, D,L-homocysteine 95% (Hcy), GSH, GSH disulfide (GSSG), fibrinogen from bovine plasma, formic acid (FA) 99%, quinine sulfate dihydrate >99%, methionine, N-acetyl-L-cysteine (N-Ac-Cys), N,N-Dimethyl-p-phenylenediamine (DMPD) 97%, phosphate-buffered saline (PBS), protamine (from Clupea pallasii), sodium citrate dehydrate, tris(2-carboxyethyl)phosphine hydrochloride (TCEP), Na_2_S·xH_2_O (≥60%), and ZnAc_2_ (Sigma-Aldrich, St. Louis, MO, USA); human serum albumin (HSA) 95% lyophilized (Reanal, Budapest, Hungary); trypsin bovine lyophilized (Samson-Med, St. Petersburg, Russia); hemoglobin (Hb) standard solution 120 g/L (Olvex Diagnosticum, St. Peterburg, Russia); collagen from calf skin (Calbiochem-Behring Corp, La Jolla, CA 92037, USA); collagen type I, 7.5 g/L, sterile (BioloT, St. Peterburg, Russia); recombinant human insulin 100 ME/mL (Actrapid^®^, Novo Nordics A/C, Mainz, Denmark); and bovine albumin (BA), heat shock isolation (Amresco, Solon, Ohio 44139, USA).

All stock solutions were prepared with distilled water filtered through 0.45 μm filters (Whatman, Marlborough, MA, USA). Stock mixtures of BSA (50 g/L), BA (50 g/L), HSA (50 g/L), and trypsin (10 g/L) were prepared in PBS. Fibrinogen (10 g/L) was dissolved in PBS with 0.1 M SDS; collagen (10 g/L), in 0.1 M HCl; and cystine (40 mM), in 0.5M HCl.

### 2.3. Human and Animal Specimens

Male outbred white rats (300–350 g) were used in the experiments. The rats were housed in Macrolin cages under controlled temperature (19–25 °C) and humidity conditions (30–70%). Food and water was available ad libitum. A light was kept on from 7:00 a.m. to 7:00 p.m.

Hyperhomocysteinemia was simulated by adding L-methionine (>99%, PanReac, Barcelona, Spain) to water at a concentration of 5 g/L (0.5%) for 14 days (*n* = 14). The average daily volume of solution consumed was 83 mL/kg body weight, corresponding to a dose of 415 mg methionine/kg per day, which is above the standard intake rate. A control group of rats (*n* = 15) was maintained under the same conditions but without the addition of methionine.

The animals were anesthetized with 50 mg/kg of pentobarbital sodium (Nembutal^®^, Ovation Pharmaceuticals, Deerfield, IL, USA) before biomaterial collection (blood and internal organs). Anesthesia depth was assessed based on the absence of a vibrissae response to a pain stimulus. An anesthetized rat was fixed in supine position. A skin incision was made over the trachea. The common carotid artery was isolated below the left sternocleidomastoid muscle, and a ligature and spatula were inserted underneath it. After fixation, an artery incision was made on the surface of the spatula using orbital scissors. The incision site was pre-irrigated with a 0.38% sodium citrate solution. After arterial incision, blood was immediately collected in a 1.5 mL tube containing 0.15 mL of 3.8% sodium citrate. Control animals were then euthanized with a double dose of anesthesia. Their internal organs (brain, liver, and kidneys) were removed, and biomaterial was immediately frozen (−74 °C).

Fasting venous blood was taken from 10 healthy volunteers (40–75 years old, 50% male) during routine examination and from 20 patients with acute ischemic stroke (49–59 years old, 70% male) who were admitted to the neurology department of the Moscow Regional Research and Clinical Institute n.a. M.F. Vladimirskiy (MONIKI) within the first 10–72 h after the onset of the neurological disorder symptoms. All participants gave informed consent for this study. The inclusion criterion for this cohort was the absence of cardiovascular pathology, blood diseases, kidney diseases, a history of oncological diseases, and use of narcotic drugs. Blood samples were collected in Lab-Vac 3 mL K3EDTA vacuum tubes (Shandong Chengwu Medical Products Factory, Heze City, China).

### 2.4. Preparation of Blood and Internal Organs

Arterial blood was centrifuged at 2000× *g* for 5 min immediately after collection. Plasma was separated, and an equivalent volume of PBS was added to the remaining blood cells (BCs). The samples were then frozen at −74 °C and stored until analysis.

Plasma and BC. After defrosting, 10 µL of plasma (in heparin) or 20 µL of BC was collected for analysis, and the sample volume was brought to 50 µL with water.

Plasma filtrate. Plasma samples were filtered through an Amicon Ultra—0.5 mL 3K Ultracel^®^ membrane (Millipore Corp, Billerica, MA, USA) at 12,500× *g* for 15 min. A total of 100 µL of filtrate was collected for analysis.

Internal organs. Organ samples (50–100 mg) were mechanically homogenized in 50 mM Na phosphate, pH 7.4, at a ratio of 1 mg tissue to 10 μL buffer. A total of 50 μL of homogenate was also collected for analysis.

Fibrin clot. To form a fibrin clot, 50 µL of 0.45 M CaCl_2_ was added to 500 µL of plasma in a 1.5 mL tube, and the mixture was incubated for 15 min at 25 °C on a shaker. The plasma clot was then removed, washed with water, and returned to the tube. A total of 0.5 mL of water was added, and the sample was centrifuged several times for 5 min at 2000× *g*, removing the supernatant and adding 0.5 mL of water. After the clot volume reached a minimum, it was washed with acetone and dried under vacuum (20 min at 30 °C); the mass of the residue (1.2–1.8 mg) was determined before analysis.

### 2.5. Cell Cultures

THP-1 cell line (ATCC collection) was cultured in RPMI-1640 media with 2 mM L-glutamine, 1 mM sodium pyruvate, and 10 mM HEPES (Paneco, Moscow, Russia) and 10% *v*/*v* Fetal Bovine Serum (Capricorn Scientific GmbH, Ebsdorfergrund, Germany). Cell cultivation was carried out in 5% CO_2_ at a temperature of 37 °C. For chronic oxidative stress, 10 μM NaAsO_2_ (Merck KGaA, Darmstadt, Germany) dissolved in PBS was added to the media. Cells were incubated for 24 h at a concentration of 1 × 10^6^ cell/mL. For chronic reductive stress, 0.75 mM DTT (Sigma-Aldrich, St. Louis, MO, USA) dissolved in PBS was added to the media. Cells were incubated for 96 h at a concentration 0.5–1 × 10^6^ cell/mL. The media was changed and supplemented with fresh DTT every 24 h. At the end of incubation, the cells were washed from the media via sedimentation (400× *g*, 5 min) and the cell pellet was frozen (−80 °C).

EA.hy926 (ATCC collection) was cultured in DMEM medium (Biolot, St. Petersburg, Russia) with high glucose content (4.5 g/L), 10% *v*/*v* Fetal Bovine Serum, 50 μg/mL gentamicin, 2 mM L-glutamine, 2 mM hypoxanthine-aminopterin-thymidine (Paneco, Moscow, Russia), and 1% MEM Non-Essential Amino Acids Solution (100×) (Life Technologies Limited, Renfrew, UK). The cell line was passaged once every 3–4 days according to the generally accepted method [34]. The cells of passages 3–13 were used in the experiments, and cells were passaged when the cultures reached 90% confluency for each line. Before freezing, the cells were washed from the nutrient medium with PBS and passaged in PBS with 5% DMSO (Sigma-Aldrich, St. Louis, MO, USA), and the cell pellet (2.5–6 × 10^6^ cells) obtained after centrifugation (850× *g*, 5 min) was stored at −80 °C.

After thawing, 0.1 mL of water and 0.2 mL of ACN were added to the cell pellet, after which the samples were vigorously mixed and centrifuged for 10 min at 15,000× *g*. The supernatant was removed, and the pellet was used for analysis.

### 2.6. ALSS

**Figure 1:**
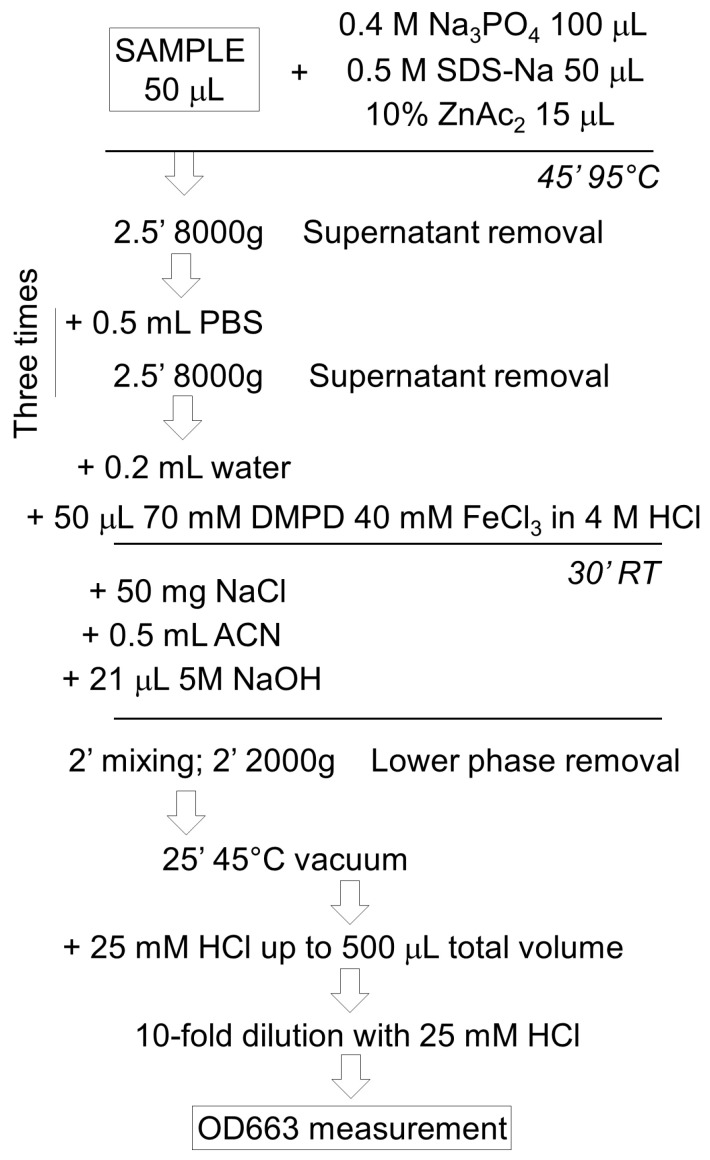
Sample preparation scheme for ALSS evaluation.

The scheme for ALSS evaluation is shown in Figure 1. In total, 100 μL of 0.4 M Na_3_PO_4_, 50 μL of 0.5 M SDS, and 15 μL of 10% (*w*/*v*) ZnAc_2_ were added to a sample in a 1.5 mL tube. The mixture was incubated for 45 min at 95 °C on a shaker. Then, it was centrifuged for 2.5 min at 8000× *g*, and the supernatant was discarded. A total of 0.5 mL of PBS was added to the pellet, after which the mixture was vigorously shaken and then centrifuged for 2.5 min at 8000× *g*. The supernatant was discarded, and the pellet washing procedure was repeated twice. Then, 0.2 mL H_2_O and 50 μL of the reaction mixture (70 mM DMPD, 40 mM FeCl_3_ in 4 M HCl) were added to the pellet and incubated for 30 min. After adding 50 mg of NaCl, the mixture was stirred until the salt dissolved, and then 0.5 mL ACN and 21 μL 5M NaOH were added to the tube. The mixture was vigorously stirred for 2 min and centrifuged for 2 min at 2000× *g*. The upper phase was collected and concentrated under vacuum for 30 min at 45 °C. The sample volume was then brought to 500 μL with 25 mM HCl.

The optical density of the samples was determined at 663 nm after 10-fold dilution with 25 mM HCl. An extract of 0.2 mL H_2_O and 50 μL of the reaction mixture was used as a blank, and extract of 0.2 mL of 0.2 mM MB and 50 μL of the reaction mixture, prepared as described above, was used as a calibrator. ALSS was calculated as the amount of MB per volume/mass of the final extract or initial sample, or as the ratio of the amount of MB to the mass of protein or the number of cells in the culture, or as the number of molecules of generated H_2_S per protein molecule (for commercial protein preparations).

### 2.7. Determination of Total Protein

The total protein content in blood plasma, culture cells, and internal organ homogenates was determined using the biuret method with the commercial reagent kit “Total protein—Agat” (Agat-Med, Moscow, Russia), according to the manufacturer’s instructions.

### 2.8. Determination of Hb Content

The concentration of Hb in BC samples was determined using the hemiglobin cyanide method with the commercial reagent kit “Hemoglobin-Olvex” (Olvex Diagnosticum, St. Petersburg, Russia), according to the manufacturer’s instructions.

### 2.9. CE and HPLC

The MB content in extracts of some model mixtures was determined via CE with UV detection, as described in [35]. Total homocysteine and cysteine content in rat blood plasma was determined via HPLC with UV detection as described previously [36].

### 2.10. Data Processing

Quantitative indicators were expressed as mean ± standard deviation or medians (1st and 3rd quartiles). A critical significance level (*p*) was calculated using a *t*-test or Mann–Whitney test for group comparisons. In the case of multiple comparisons, the Holm–Bonferroni method was used to correct *p* values.

## 3. Results

### 3.1. H_2_S Generation from Protein Under Alkaline Conditions

**Figure 2:**
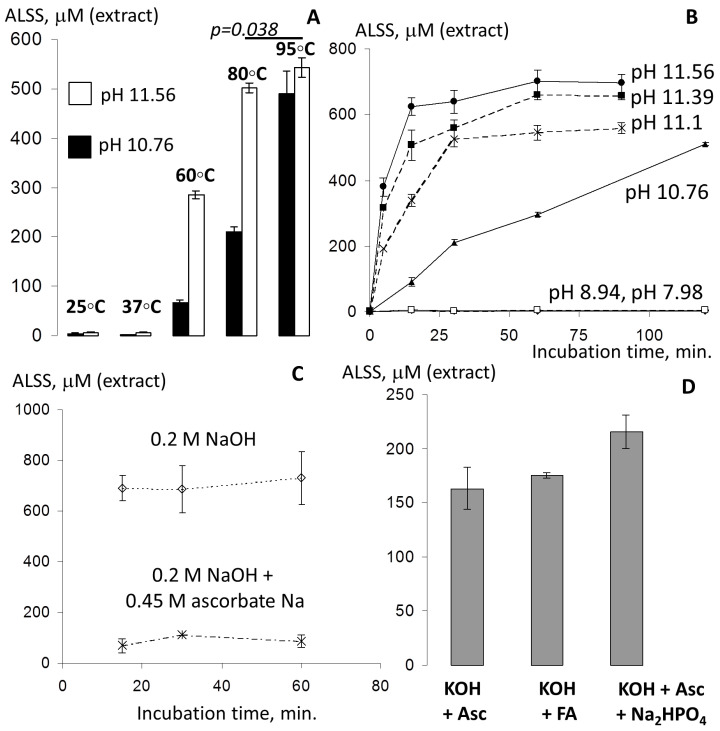
(**A**) The effect of temperature on the release of H_2_S from BSA in 0.2 M Na phosphate buffers (30 min incubation). (**B**) The effect of pH and incubation time on the release of H_2_S from BSA in 0.2 M Na phosphate buffers. (**C**) H_2_S released from BSA in NaOH with/without ascorbate Na. Incubation mixtures (6.25 g/L BSA in the buffer with 0.125 M SDS and 0.7% ZnAc_2_; total volume: 215 µL) were prepared as described in Section 2.6, at t = 80 °C. (**D**) The influence of ascorbic acid on the release of H_2_S from BSA in the incubation mixture (37 °C, 3 h): “KOH+Asc”: 6.65 g/L BSA in 3 M KOH with 0.9 M ascorbic acid; “KOH+Asc”: ascorbic acid was replaced with FA; “KOH+Asc+Na_2_HPO_4_”: Na_2_HPO_4_ (0.4 M) was added to BSA/KOH/ascorbic acid mixture (*n* = 3).

We investigated the effect of temperature on the H_2_S yield using model mixtures (12.5 g/L BSA and 0.125 M SDS in 0.2 M Na-phosphate buffer with pH 10.76 or 11.56), which were incubated for 30 min in the presence of ZnAc_2_ (0.7% *w*/*v*) followed by the washing of the precipitate, MB synthesis, and its salting out, as described in Section 2.6. As shown in Figure 2A, the incubation of the model mixtures at 25 °C and 37 °C was not accompanied by a significant H_2_S yield; however, at 80–95 °C, a significant increase in H_2_S yield was observed. The effect of pH and incubation time on H_2_S yield was then investigated using the same model mixtures incubated in Na-phosphate buffers with pH from 7.98 to 11.56, also in the presence of Zn and SDS, as described above, but at 80 °C. Figure 2B shows that under mild conditions (pH ~8–9), the incubation of the mixture for two hours is practically not accompanied by the generation of H_2_S. Further, with increasing pH, both the rate of H_2_S release and its concentration in the mixture increase. Of the buffers studied, 0.2 M Na_3_PO_4_ (pH 11.56) was chosen as the optimal incubation buffer. When using 0.2 M NaOH under the same conditions (Figure 2C), H_2_S yield was approximately at the same level (*p* = 0.738 at 60 min of incubation); however, the variability of H_2_S yield when using NaOH was significantly higher than when using Na_3_PO_4_. When Na ascorbate (0.45 M, pH 9.0) was added to the incubation mixture containing 0.2 M NaOH, the H_2_S yield underwent a dramatic drop (Figure 2C).

The effect of ascorbic acid on the yield of H_2_S from BSA (6.65 g/L) was also studied under the incubation conditions given in [25] (3 M KOH, 0.9 M ascorbic acid, 37 °C, 3 h). Figure 2D shows that replacing ascorbic acid with FA does not lead to a significant change in H_2_S yield, while the addition of Na_2_HPO_4_ (0.4 M) to the mixture significantly increases it.

By varying the amount of BSA (from 125 to 2500 µg) in the incubation mixtures, the optimal sample amount for analysis was determined. In the range up to 1250 µg BSA, no significant differences in H_2_S generation from BSA were observed, but sample overload was observed at 2500 µg BSA (Figure S1).

Using model mixtures of 10 g/L BSA in PBS, processed as described in Section 2.6, the dependence of H_2_S generation on incubation time (15–90 min) at 95 °C was studied. No significant differences were found in the 30–90 min range, so 45 min was chosen as the optimal incubation time under these conditions (Figure S2). Replacing Na_3_PO_4_ with K_3_PO_4_ under the same conditions (but without SDS) caused a noticeable decrease in ALSS to 64.9 ± 8.9% (Figure S3).

The recovery of H_2_S precipitation in the form of ZnS was calculated by determining the H_2_S content in the supernatant of model mixtures containing 50 mg/L Na_2_SxH_2_O (≥60%) in PBS (*n* = 3), which were incubated with ZnAc_2_, as described in Section 2.6 (“Na_2_S+ZnAc_2_”). The samples were then centrifuged (2.5 min, 8000× *g*), and the supernatant was collected, to which a reaction mixture containing DMPD and FeCl_3_ in HCl was added, followed by MB salting-out as described in Section 2.6. To “control” samples (*n* = 3) with the same amount of Na_2_S, without ZnAc_2_ or SDS, this reaction mixture was added without preliminary incubation, and MB salting-out was carried out as described above. For the “blank” series samples (*n* = 3), PBS without Na_2_S was used, and these samples were processed in the same manner as those of “Na_2_S+ZnAc_2_” series. The resulting extracts were analyzed via CE as described in Section 2.8. Table S1 presents the MB concentrations in these samples. In the presence of ZnAc_2_, the total H_2_S in the model mixture achieves almost complete precipitation: 97.8 ± 2.8%.

**Figure 3:**
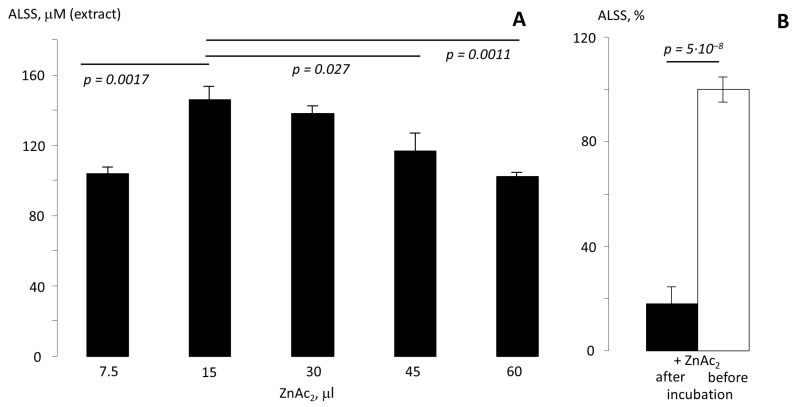
(**A**) Influence of ZnAc_2_ amount (10%) on the yield of H_2_S from a model BSA mixture (50 µL, 10 g/L) treated as described in Section 2.6 (*n* = 3). (**B**) Effect of prior and delayed addition of ZnAc_2_ to the BSA model mixture (50 µL of 10 g/L) on the ALSS evaluation (*n* = 4).

We also investigated the effect of ZnAc_2_ amount (10% *w*/*v*, 7.5–60 μL) on H_2_S yield in the model BSA mixture (50 μL of 10 g/L) treated as described in Section 2.6 (Figure 3A). As shown in Figure 3A, the optimal amount of 10% ZnAc_2_ is 15–30 μL, which corresponds to 0.7–1.4% (38–76 mM) in the incubation mixture. In addition, a comparative study was conducted between the preliminary and delayed addition of ZnAc_2_ to the incubation mixture with blood plasma. One series (*n* = 4) was processed as described in Section 2.4 and Section 2.6, while in the second series (*n* = 4), ZnAc_2_ was added after 45 min incubation of samples at 95 °C. As a result, the ALSS of the second series was only 17.9 ± 6.1% of the level of the first series (Figure 3B), indicating a significant loss of H_2_S during the thermal processing of samples under alkaline conditions in the absence of Zn^2+^.

### 3.2. MB Synthesis and Extraction

We searched for the optimal concentrations of HCl, DMPD, and FeCl_3_ for MB synthesis in a model mixture containing 20 mg/L Na_2_SxH_2_O. Initially, MB production was studied in a mixture with a fixed concentration of DMPD (8.7 mM) and FeCl_3_ (13 mM) and different HCl concentrations (0.25–1.5 M). After 30 min of incubation, the samples were diluted 10-fold with 20 mM HCl, and their optical density was determined at 663 nm. The highest absorbance was observed at 0.75–1 M HCl (Figure S4A). Then, we investigated the effect of different concentrations of FeCl_3_ (4–24 mM) in mixtures containing 5 mg/L Na_2_SxH_2_O and 8.7 mM DMPD in 0.8 M HCl. The samples were diluted three times with water after incubation. In this case, the highest absorption signal corresponded to a FeCl_3_ concentration of 8 mM in the mixture (Figure S4B). Finally, to select the optimal DMPD concentration, we added DMPD (2–24 mM) to mixtures containing 10 or 0 (control) mg/L Na_2_SxH_2_O, 0.8 M HCl, and 0.2 M FeCl_3_. The samples were diluted 10-fold with water after incubation, and the highest absorbance was observed at 14 mM DMPD in the mixture (Figure S4C).

**Figure 4:**
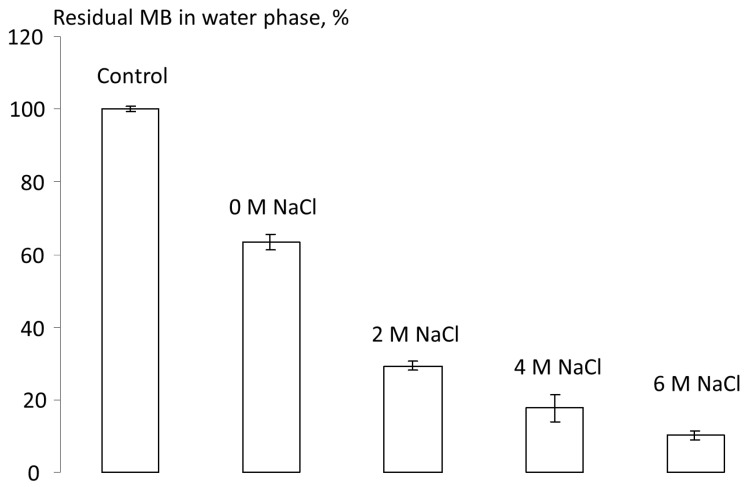
Effect of NaCl content on the efficiency of MB salting-out. Mixture composition: 100 μL 0.5 mM MB, 100 μL NaCl solution, 50 μL 4 M HCl, 21 μL 5M NaOH, and 500 μL ACN (*n* = 3).

To purify MB, we investigated the conditions for its salting-out in the organic phase. The effect of NaCl concentration on the efficiency of MB salting-out was studied using model mixtures containing MB (0.5 mM, 100 μL), HCl (4 M, 50 μL), NaOH (5 M, 21 μL), ACN (500 μL), and NaCl (0–6 M, 100 μL). Water was added to the control mixtures instead of acetonitrile and NaCl. After mixing the samples, the upper (ACN-rich) phase was discarded, and the MB content in the aqueous phase was determined. As shown in Figure 4, with increasing NaCl concentration, the MB content in the aqueous phase decreased. Thus, at a NaCl concentration in the mixture of 175.5 g/L (“6M NaCl”), only 10.2 ± 1.3% of the total MB remained in the aqueous phase. Then, with the samples of these model mixtures prepared as described in Section 2.6, we calculated that the efficiency of MB salting-out (with subsequent vacuum concentration of the organic phase and re-dissolution of the residue) is 89.7 ± 1.2% (*n* = 4).

During the oxidation of DMPD, byproducts of its polymerization are also formed, which interfere with MB. Figure S5 shows the absorption spectrum of the reaction mixture (14 mM DMPD, 8 mM FeCl_3_ in 0.8 M HCl) without the addition of Na_2_S, in which a broad absorption peak in the region of 535–550 nm is distinguished. The shoulder of the peak also extends into the region of MB detection (663 nm), where the extinction level drops by a factor of 5 relative to the maximum (540 nm). When salting-out this model mixture, the intensity of the absorption signal at 663 nm in the ACN-phase extract decreased by a factor of 8.52, and the intensity of the absorption signal in the aqueous extract was 90.6% relative to the initial mixture and their volumes. Thus, salting-out made it possible to reduce the level of background absorption by an order of magnitude.

### 3.3. Reduction and Desulfurization of BSA Polysulfides

**Figure 5:**
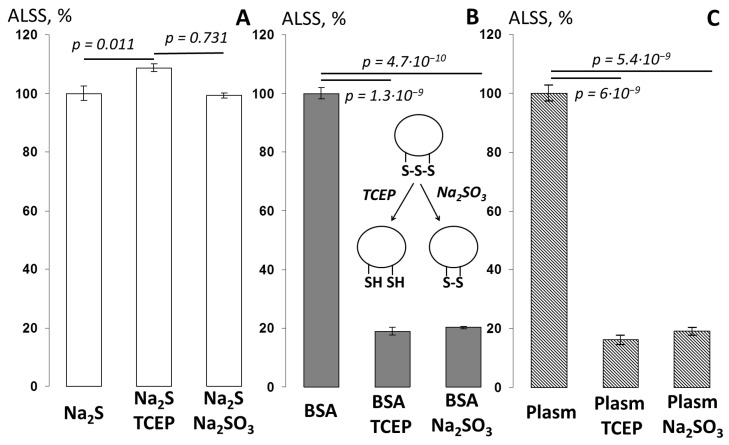
(**A**) Effect of TCEP and Na_2_SO_3_ (0.1 M, 50 μL) on ALSS of Na_2_SxH_2_O (100 mg/L, 50 μL). (**B**) Effect of pre-incubation (30 min, 37 °C) of BSA (10 g/L, 50 μL) with TCEP and Na_2_SO_3_ (0.1 M, 50 μL). (**C**) Effect of pre-incubation (30 min, 37 °C) of blood plasma (10 μL) with TCEP and Na_2_SO_3_ (0.1 M, 50 μL). Samples were processed as described in Section 2.6 (*n* = 4).

To assess the method’s specificity for sulfides, we investigated the effects of two approaches to (hydro)polysulfide degradation. The first relies on the reducing action of TCEP, and the second on the desulfurizing action of sulfite (Figure S6). We first ensured that the presence of TCEP and Na_2_SO_3_ did not have a negative effect on the formation of ZnS precipitate. The incubation of the model mixture containing 100 mg/L Na_2_S xH_2_O (≥60%), as described in Section 2.6, with the addition of 50 µL 0.1 M TCEP or Na_2_SO_3_ resulted in a slight increase in the amount of MB (by 8.7%, *p* = 0.011) in the first case, and had no effect on the product yield in the second (Figure 5A). Then, BSA samples (50 μL, 10 g/L) were pre-incubated for 30 min at 37 °C with the addition of 50 μL 0.1 M TCEP or Na_2_SO_3_ and were processed as described in Section 2.6. The ALSS of these samples in both cases was significantly reduced, amounting to about 19.1% (TCEP) and 20.2% (Na_2_SO_3_) compared to the control samples containing no TCEP or Na_2_SO_3_ (Figure 5B). In a similar manner, the incubation of a human plasma mixture in the presence of TCEP and Na_2_SO_3_ resulted in a decrease in ALSS to 16.2% and 19.1%, respectively (Figure 5C). Thus, both approaches to polysulfide degradation yielded similar results for both the BSA model mixture and plasma. The specificity of the developed approach is approximately 80%.

### 3.4. Sensitivity, Linearity, Stability, and Reproducibility of the ALSS Evaluation Method

The detection limit (S/N = 3) of ALSS for the photometric detection of extracts was 1 nmol, and the limit of quantification (S/N = 10) was 3.3 nmol of released H_2_S in the sample.

For model mixtures in the Na_2_SxH_2_O (≥60%) concentration range of 0.125–4 mg/L, prepared as described in Section 2.6 and analyzed using the CE method as specified in Section 2.8, a linear dependence of the signal on the analyte concentration was observed over the entire studied range (MB/Internal Standard = 53.5 × Na_2_S (mg/L) + 3.06, r^2^ = 0.9979). Linearity for model MB mixtures (12.5–400 μM), to which the reaction mixture was added and for which salting-out was performed as described in Section 2.6, was also observed over the entire studied range (OD^663nm^ = 0.0018 × MB (μM) + 0.0032, r^2^ = 0.9999).

The optical absorbance of Na_2_SxH_2_O model mixture extracts (100 mg/L) remained stable for two days of storage at 25 °C in the dark and then gradually decreased to ~83% after two weeks of storage (Figure S7A). This was likely due to MB adsorption on the test tube walls. When storing the blood plasma mixture (37 °C in the dark, *n* = 4), the ALSS level increased by 21% (*p* = 6 × 10^−5^) within 4 h, but no level difference was observed after 24 h (99.2%, *p* = 0.97); see Figure S7B. After 4 days of storing the plasma under these conditions, the ALSS level decreased to 93% (*p* = 0.016). Repeated freeze-thawing of plasma stored at −80 °C did not significantly affect ALSS (Figure S7C).

The interday reproducibility of the method was determined using mixtures of blood plasma, plasma filtrate, and BC (*n* = 3), which were independently prepared and analyzed for three days as described in Section 2.4 and Section 2.6. The corresponding CVs were 5.71% (plasma), 6.15% (plasma filtrate), and 4.47% (BC). The intraday reproducibility was 3.6% (2.0–5.6; plasma), 6.1% (2.2–8.8; plasma filtrate), and 1.9% (0.6–3.7; BC).

### 3.5. Evaluation of ALSS in Low-Molecular-Weight Aminothiols and Ammonium Polysulfide Solution

To evaluate ALSS in commercial preparations of low-molecular-weight aminothiols (Cys, N-Ac-Cys, methionine, cysteinylglycine, GSH, and GSSG) and Na_2_S_2_O_3_, 1 mM mixtures of these substances were prepared in PBS and processed as described in Section 2.6. The yield of H_2_S was not reliably detected in Cys and N-Ac-Cys (Table 1). The H_2_S/thiol ratio was <5% for cysteine, methionine, cysteinylglycine, and thiosulfate. This value for GSH (6.5%) and especially for GSSG (19.9%) turned out to be significantly higher, which indicates significant generation of H_2_S from these compounds under alkaline conditions upon heating.

To evaluate ALSS in ammonium polysulfide solution, it was diluted 1000-fold and also processed as described in Section 2.6. Unlike low-molecular-weight aminothiols, the polysulfide extract was characterized by a high yield of H_2_S, since the ALSS of the preparation was 6.39 ± 0.38 M.

### 3.6. ALSS of Protein Preparations

ALSS levels in commercial protein preparations are given in Table 2. For albumins, ALSS was approximately 18 molecules of released H_2_S per protein molecule (i.e., more than 0.5 atoms of sulfane sulfur per cysteine residue). Other secretory proteins, such as fibrinogen, trypsin, and insulin, also had relatively high ALSS levels (~0.36 to ~0.48 sulfonic sulfur atoms per cysteine residue). This value for hemoglobin was significantly lower. Proteins lacking cysteine residues (collagen and protamine) had very low ALSS levels (<5 μmol/g).

### 3.7. Evaluation of ALSS in Blood and Internal Organs

**Figure 6:**
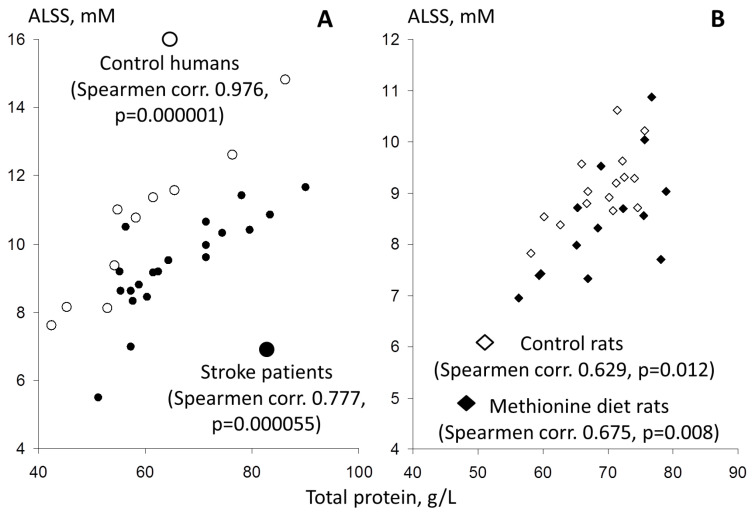
Association of ALSS and total plasma protein concentrations in human (**A**) and rat (**B**) blood plasma samples.

Table 3 presents the results of the ALSS evaluation of the plasma of healthy volunteers, stroke patients, and rats. In these groups, ALSS was approximately 7.5–11.5 mM; however, due to the lower total protein content, ALSS per unit protein mass was significantly higher in humans than in rats. These groups showed a characteristic association between ALSS and total plasma protein concentrations, the strongest being observed in human plasma (Figure 6A). Although the plasma ALSS level in the stroke group did not differ significantly from the control group in absolute concentration (*p* = 0.231), this indicator, normalized per unit mass of total protein, was significantly lower (*p* = 4 × 10^−5^, Table 3). The total homocysteine level in the patient group was also significantly higher than in the control group (17.8 (15; 22) vs. 12 (11.7; 12.3) μM, *p* = 0.001). In the combined group of controls and patients, an inverse association of ALSS (μmol/g protein) and homocysteine levels was observed (Spearmen r = −0.518, *p* = 0.003).

Analysis of eight rat BC samples showed that the ALSS was 5.16 (4.76; 5.66) mM, but when considering the Hb content, ALSS was 52.6 μmol/g Hb (41.8; 55.9). No significant association of ALSS with Hb concentration was found. In rat plasma filtrates (*n* = 4), ALSS was—184.8 (from 158 to 235) μM. The ALSS of fibrin clot (7 samples) was 70.5 (65; 71.2) μmol/g, which is close to that of fibrinogen (65.2 μmol/g).

The results of the ALSS evaluation in rat internal organ homogenates are shown in Table 4. In terms of protein mass, ALSS in the heart and brain was similar (*p* = 0.9), but due to the lower protein content, ALSS per unit of tissue mass in the brain was significantly lower than in the heart (*p* = 0.006). ALSS in the liver was higher than in the brain and heart (*p* = 0.018), and ALSS in all internal organs was significantly lower than in blood plasma.

### 3.8. Evaluation of ALSS in Cell Culture and the Effect of Oxidative/Reductive Stress

**Figure 7:**
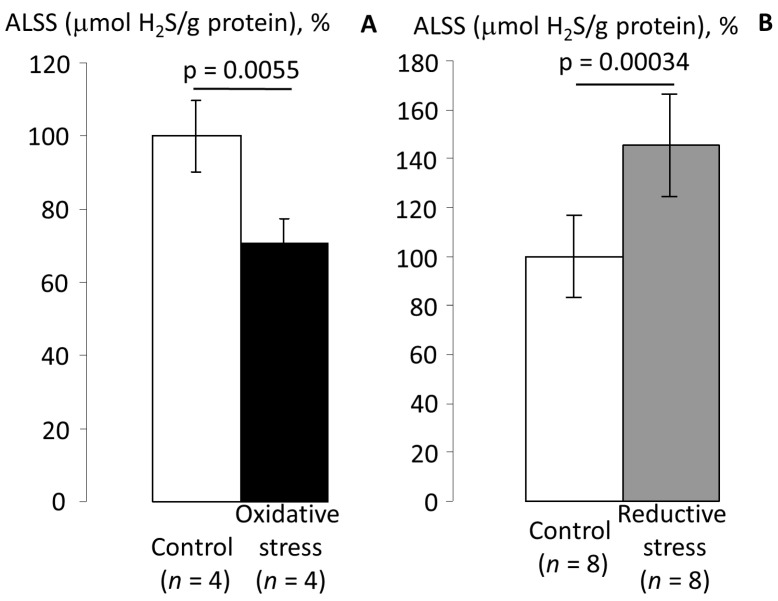
Effect of chronic oxidative (**A**) and reductive (**B**) stress on ALSS in THP-1 culture.

ALSS was 4.73 ± 1.02 nmol/10^6^ cells or 22.2 ± 4.8 μmol/g protein in the endothelial cell culture Ea.hy926. We also examined the effects of chronic oxidative stress (in the presence of sodium arsenide; 24 h) and chronic reductive stress (in the presence of dithiothreitol; 4 days) on ALSS in the THP-1 monocytic cell culture. Under normal conditions, ALSS in the extract of this culture was 16.06 ± 0.86 nmol/10^6^ cells (14.8 ± 1.3 μmol/g protein) after 24 h incubation, while under oxidative stress, this value decreased to 9.22 ± 0.48 nmol/10^6^ cells (10.4 ± 1.0 μmol/g protein), *p* = 1.5 × 10^−5^ and 0.0055, respectively (Figure 7A). Moreover, no significant decrease in total protein was detected in these cells under the influence of arsenide (control: 1.1 ± 0.15; stress: 0.9 ± 0.12 mg/10^6^ cells, *p* = 0.116). Under chronic reductive stress, no significant changes in ALSS cells were observed compared to the control group (control: 12.6 ± 2.8; reductive stress, 11.8 ± 2.6 nmol/10^6^ cells, *p* = 0.64); however, the protein level in the cells was reduced (control: 1.14 ± 0.12; reductive stress: 0.734 ± 0.075 mg/10^6^ cells, *p* = 8 × 10^−6^). As a result, ALSS per unit mass of protein was increased (control: 10.9 ± 0.9; reductive stress: 15.9 ± 2.0 μmol/g protein, *p* = 0.00034) as shown in Figure 7B.

### 3.9. The Effect of a Methionine Diet on ALSS in Blood Plasma in Rats

In rats fed a methionine diet, the level of total Hcy in blood plasma was significantly increased (diet: 9.88 (7.33; 15.06) μM; control: 3.54 (3.21; 3.69) μM; *p* = 10^−7^) and an increase in the level of total Cys was also observed (diet: 121.4 (110.4; 136.2) μM; control: 105.2 (91.6; 108.0) μM; *p* = 0.002). The results of the ALSS evaluation in the blood plasma of rats fed a standard diet and a diet with a high content of methionine (to create a mild hyperhomocysteinemia) are also presented in Table 3. As can be seen in this table, there are no reliable changes in the concentration of ALSS and protein content in the plasma of rats against the background of methionine consumption. However, although the ALSS content per unit of total protein mass decreased slightly, it was noticeable (up to 92% of the control, *p* = 0.036). The methionine diet group also showed an association between ALSS and total plasma protein concentrations (Figure 6B).

### 3.10. Matrix Effect (Influence of Plasma Filtrate on ALSS of BSA)

**Figure 8:**
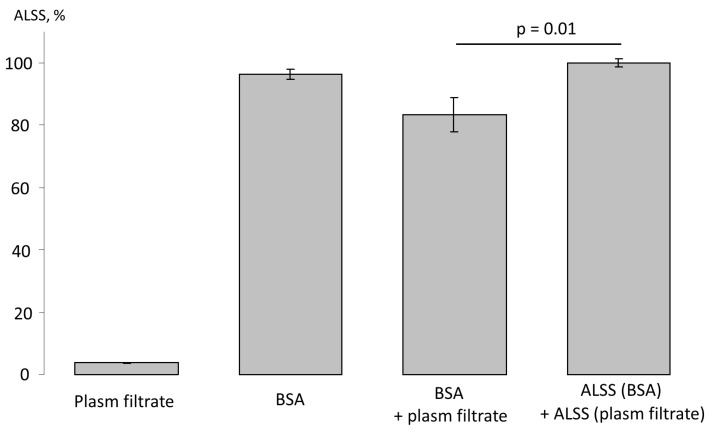
Effect of blood plasma filtrate on ALSS of a model mixture with BSA (*n* = 4).

To assess the possible matrix effect on ALSS, 40 µL of PBS or plasma filtrate was added to 10 µL of 50 g/L BSA, and the samples (*n* = 4) were prepared as described in Section 2.6. As shown in Figure 8, if the sum of the ALSS of plasma filtrate and BSA is taken as 100%, then in their mixture, the ALSS will only be 83.3 ± 5.4%, while in BSA samples without filtrate, the ALSS was 96.2 ± 1.6%. Thus, the addition of plasma filtrate reduces ALSS, indicating the presence of a matrix effect.

The addition of an equivalent volume of aminothiols (Cys or cystine) at a high concentration (5 mM) to the BSA sample (50 µL, 10 g/L) resulted in a decrease in ALSS to ~88% of the control level, while when the same volume of 2.7% H_2_O_2_ was added to the incubation mixture, ALSS dropped to almost zero (Figure S8).

## 4. Discussion

**Figure 9:**
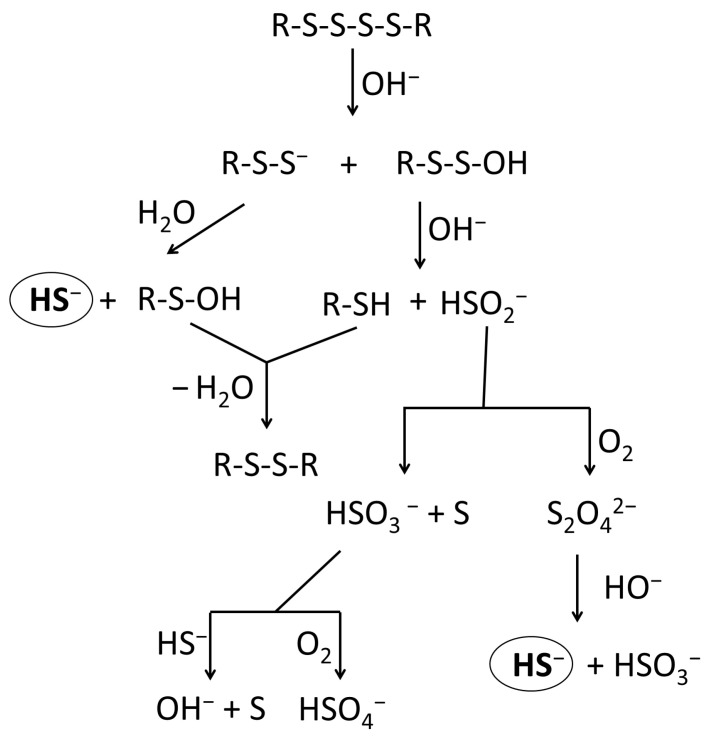
Proposed mechanism of alkaline hydrolysis of polysulfide chains in proteins.

While maintaining the general principles of the previously proposed EMSP-MB method, we examined the conditions for H_2_S generation from the model protein mixture in more detail and introduced a number of significant modifications. The authors of the original EMSP-MB method [26] assumed that ascorbic acid plays a significant role in H_2_S generation from proteins under alkaline conditions, acting as a reducing agent. However, our results show that the presence of ascorbic acid is not required; it can be replaced under the experimental conditions of EMSP-MB with equal efficiency, for example, with formic acid, which does not possess its own reducing action. Conversely, the incubation of the BSA mixture at 80 °C revealed that the presence of ascorbate can significantly reduce H_2_S yield. This suggests that ascorbic acid is not involved in the mechanism of the alkaline hydrolysis of polysulfides and rather plays a buffering role. Then, since the release of H_2_S is observed even in the absence of reducing agents in the incubation mixture, the mechanism of alkaline hydrolysis of polysulfides is apparently based on the disproportionation of sulfane sulfur atoms (Figure 9). According to this mechanism, a molecule of sulfoxylic acid (S^+2^) should be formed per hydrogen sulfide molecule (S^−2^). However, sulfoxylic acid is metastable and quickly decomposes into sulfur (S^0^) and sulfurous acid. The latter can also form sulfur in a reaction with H_2_S or be oxidized to sulfate by oxygen dissolved in the sample. Sulfoxylic acid can also be oxidized by oxygen to dithionite (S_2_O_4_^2−^), which, in turn, can also disproportionate into sulfite and sulfide [37]. Thus, in biological matrices with their complex composition, due to the many reactions, it is very difficult to determine the actual yield of H_2_S; therefore, it is very difficult to accurately determine the amount of sulfane sulfur bound to proteins. This also clearly confirms that in the presence of blood plasma filtrate, a decrease in H_2_S release from BSA was observed, i.e., a matrix effect. Therefore, the proposed approach is not a method for determining sulfane sulfur, but rather a way to evaluate this pool in biological samples.

The formation of H_2_S from protein polysulfides is significantly affected by both the temperature and pH of the incubation mixture. Under slightly alkaline conditions (pH < 9), H_2_S is virtually not formed even upon heating, while at pH 11~11.6, the rate of H_2_S generation increases significantly. In the EMSP-MB method, the authors preferred KOH over NaOH, since the H_2_S yield was higher when BSA was incubated in a KOH mixture at 37 °C than when incubated in NaOH. However, when the model BSA mixture was incubated at 95 °C in Na triphosphate, ALSS was significantly higher than when using K triphosphate, which was likely due to a decrease in ZnS formation in the presence of a high K^+^ concentration, rather than a decrease in H_2_S generation. Visually, model BSA mixtures containing K triphosphate or water were significantly more transparent than mixtures to which Na triphosphate was added, indicating the significant ability of albumin to adsorb Zn^2+^ under these conditions, thereby reducing the concentration of free Zn^2+^.

Since intense heating of the sample can cause protein precipitation, which can subsequently negatively impact the analysis results (e.g., due to MB sorption in the protein precipitate), we decided to add the anionic detergent SDS to the incubation mixture, which prevents protein precipitation. Since SDS forms an insoluble precipitate in the presence of high concentrations of K^+^, this was another reason for choosing Na-phosphate buffer.

Another difference between this approach and EMSP-MB is that we added ZnAc_2_ immediately before the incubation of the mixture, rather than after, since the delayed addition of this binding agent caused significant losses of H_2_S under the new incubation conditions.

The isolation and purification of ZnS significantly improve the selectivity of H_2_S determination through the synthesis of MB from it. However, in addition to the formation of the target product, DMPD oxidation/polymerization byproducts are also formed in the reaction mixture, which increase the background absorption level and thereby reduce the selectivity and sensitivity of the method. To purify MB, we used liquid-phase extraction (LPE) based on the salting-out effect. Typically, LPE methods for MB purification are based on the addition of a highly toxic organic solvent (methylene chloride and xylene) and an ion-pair reagent (organic acids) to the sample [38,39,40], but salting-out allows us to avoid this and simplify the subsequent re-dissolution of MB in the aqueous phase. The efficiency of the salting-out and purification of the extract from byproducts was about 90%, which is comparable to that of traditional methods of LPE MB.

The photometric method for determining H_2_S through the formation of MB is not highly sensitive in itself and is used primarily for samples containing H_2_S at the level of tens to hundreds of µM [16,23]. The authors of [26], who created the EMSP-MB method, do not report the detection limit; however, based on the feasibility of saliva analysis, the LOQ of this approach was <10^−4^ M. Our approach, due to MB purification, allowed us to increase the sensitivity several times further, to 3 × 10^−5^ M (with a matrix volume of 0.1 mL). In any case, this is more than sufficient for analyzing blood plasma, and this approach has the potential to increase in sensitivity when analyzing other matrices.

Under alkaline conditions, hydrolysis of the C-S bond is possible [41], as well as, accordingly, the generation of H_2_S by cysteine residues of proteins and low-molecular-weight aminothiols present in biological samples. The ALSS evaluation results show that some of these compounds (Cys, methionine, and cystine) practically do not release H_2_S under the developed incubation conditions, and cysteinylglycine and GSH release it in small amounts, which are unlikely to have a noticeable effect given the concentration of these metabolites in blood plasma (10^−6^–10^−5^ M). The GSH content in blood cells is already ~10^−3^ M, which makes its contribution to ALSS about 1~1.5%. These results are close to those previously obtained using the EMSP-MB [26]. At the same time, GSSG demonstrated a significant ability to generate H_2_S, which cannot be attributed to possible impurities in the substance. On the one hand, the GSSG content in plasma and blood cells is only a few μM, making its contribution to ALSS insignificant. On the other hand, this suggests that some cystine or cysteine residues in proteins and peptides can also undergo significant alkaline hydrolysis, limiting the specificity of the approach.

To determine the specificity of the developed approach, we used two known methods for eliminating polysulfides. The first is based on their reduction in TCEP with the formation of H_2_S [42], and the second is based on desulfurization under the action of Na_2_SO_3_ and proceeds with the formation of thiosulfate [43]. These agents themselves had no effect on ZnS formation, but the pre-incubation of BSA with these agents resulted in a four-fold decrease in ALSS. Thus, two independent methods demonstrated that the specificity of ALSS assessment on this model mixture is at least 80%. Apparently, the mechanism of transformation of disulfides to persulfides, which was identified for GSSG [44] followed by alkaline hydrolysis of these persulfides, plays an important role in the generation of the remaining H_2_S. Note that the efficacy of these agents against albumin polysulfides may vary, and not all of them may be available for reduction or desulfurization. Specificity may differ for other proteins, but it is unlikely to be significant.

The formation of protein-bound polysulfides is still poorly understood. It is assumed that per- and hydropolysulfides of cysteine residues are initially formed through their exchange reactions with CysSSH, GSSH, or H_2_S_n_ [10,14,45], and the metabolite 8-nitro-cGMP is likely involved [46]. Enzymes such as cystathionine-β-synthase (CBS), γ-cystathionase (CSE), glutathione reductase, ethylmalonic encephalopathy protein 1 (ETHE1), persulfide dioxygenase, and 3-mercaptopyruvate sulfurtransferase (MST) are involved in the generation of per- and hydropolysulfides, while Cys, GSH, and H_2_S act as carriers of sulfane sulfur to proteins. Cysteinyl-tRNA synthase is also capable of generating CysSSH and activating the corresponding tRNA, which leads to the incorporation of this persulfide into the polypeptide chain during translation [24]. The ability to catalyze the oxidation of H2S to per- and hydropolysulfides (H_2_S_3_–H_2_S_5_) was identified in superoxide dismutase (SOD) [47]. The mechanism of oxidation of hydropolysulfides to protein polysulfides remains unclear [46]. Various studies have found that per/polysulfidation affects the activity of proteins (ETHE1, Glyceraldehyde 3-phosphate dehydrogenase (GADPH), SOD, Transient Receptor Potential Ankyrin 1 (TRPA1), NMDA receptors, actin, tubulin, alcohol dehydrogenase 5, etc.) [10,14,45,46]. Thioredoxin/thioredoxin reductase, thioredoxin-related protein, GSH reductase, and β-NADH appear to play an important role in the elimination of polysulfides [14,33].

Among the proteins studied, albumin had the highest ALSS to cysteine residue ratio (>0.5), while insulin had a slightly lower ratio (~0.48). Other extracellular proteins (fibrinogen and trypsin) were also characterized by a noticeable content of sulfane sulfur (ALSS ~0.35~0.38 per Cys residue). Hb had significantly lower levels of this indicator (~0.15). Mature collagen types I-III do not contain cysteine residues and were characterized by a low ALSS (<5 μmol/g), which was apparently due to the presence of collagen type IV, impurities, and immature forms of collagen.

Our results show that the ALSS of HSA is 18.4 sulfane sulfur atoms per molecule, and when taking into account the specificity of the method, it was 14.7. In total, albumin contains 17 intramolecular disulfide bonds, i.e., for each bond there is, on average, 0.86 atoms of sulfane sulfur, which has been reduced to H_2_S (excluding the sulfur oxidized in the process). Almost nothing is known about the distribution of the length of these polysulfide chains, but it has recently been discovered that some cystine residues of BSA are converted to a reduced form or to a persulfide form under the influence of UV irradiation, which convincingly indicates the presence of sulfane sulfur in their structure [33].

The close association of ALSS levels with the total protein content in blood plasma indicates that the overwhelming majority of H_2_S generated during the sample incubation stage is of protein origin. The ALSS of human plasma is ~11 mM, which is slightly higher overall, but close to previously obtained results [26]. The average plasma albumin content in humans is 35–50 g/L (0.5–0.725 mM), which gives a theoretical contribution of albumin to the ALSS of plasma of about 9.2–13.3 mM. However, although albumin is a Cys-rich protein (6% Cys of the total amino acid residues), it accounts for about 50–60% of the total plasma protein. If we assume that the frequency of Cys residues in other proteins is 2.26% [48] and they are loaded with sulfane sulfur to the same extent as albumin, then the theoretically expected plasma ALSS will be 11.5–18.3 mM, which is higher than the experimentally obtained estimate (10.9 mM). This also suggests that, due to the matrix effect, plasma components (thiols, reactive nitrogen, or oxygen species) trap released H_2_S under alkaline conditions or are able to compete with Zn^2+^ for H_2_S binding. For example, high concentrations of H_2_O_2_ (2.7%) completely inhibited ZnS formation. High concentrations (5 mM) of cystine and cysteine also resulted in a slight decrease in ALSS BSA, although they were sources of H_2_S under incubation conditions. Considering the concentration of these metabolites in blood plasma (reduced Cys ~15 μM and cystine ~50 μM), their contribution to ALSS can hardly be considered significant. The matrix effect of blood plasma filtrate, leading to a slight decrease in ALSS BSA, is apparently due to the combined effect of many compounds.

Fibrinogen is a key protein for thrombus formation and the primary target of thrombolysis. Disulfide bonds within and between its chains play a crucial role in stabilizing its structure [49]. Some of these bonds are labile, and 10–50% of them are normally in a reduced state [50]. However, it is still unclear to what extent their modifications can affect the properties of fibrin fibers and whether the reduction in fibrin disulfide bonds can be used as an effective approach for fibrinolysis. Our results demonstrate that the content of sulfonic sulfur in fibrinogen is quite significant, suggesting that some of its Cys residues or covalently attached homocystamide may carry polysulfide chains, the functional significance of which is still unclear.

The ALSS levels in BC are also significant (~5.2 mM or ~52 μmol/g Hb). Hb is the dominant blood protein; however, since the ALSS of Hb itself is significantly lower (13.4 μmol/g), Hb is not the main source of the sulfane sulfur pool, and no reliable correlation was found between ALSS and Hb concentration. Therefore, there is no certainty that normalizing H_2_S yield to Hb concentration is rational for ALSS evaluation; it is possible that the erythrocyte content or erythrocyte volume would be more appropriate.

Interestingly, ALSS was also detected in the plasma filtrate at a relatively high level (~185 μM), which is many times higher than the pool of free low-molecular-weight aminothiols in plasma. A drawback of ultrafiltration is the potential for a small fraction of proteins to penetrate the filtrate, so other approaches, such as protein precipitation with organic solvents or acids, may be useful in future studies. Nevertheless, the obtained result provides a reason for a more detailed study of the sulfane sulfur from low-molecular-weight thiols and peptides in plasma.

In this study, the ALSS levels in internal organs (brain, heart, and liver) were also evaluated for the first time. Although these values were significantly lower than in blood plasma, they still indicate the presence of a high content of sulfane sulfur in the tissues. Taking into account the density of these organs (1.03–1.06 g/mL), the estimated sulfane sulfur pool is 8.15–16.5 mM, which significantly exceeds the average content of the main intracellular low-molecular-weight thiol, glutathione (~1 mM), and low-molecular supersulfides (submillimolar to millimolar levels [14]). Taking into account the protein content, the ALSS of these internal organs is ~45–60 μmol/g, which is also significantly higher than that of the DTT-labile H_2_S pool (0.2–6 μmol/g [10]).

A relatively high level of ALSS (~4~17 nmol/10^6^) was also detected in cell cultures (Ea.hy926 and THP-1). The intracellular concentration of sulfane sulfur is about 2.7 and 29.5 mM in cells when taking into account their volume (Ea.hy926, 1600 fl [51]; THP-1, 580 fl [52]). The biological role of protein-bound sulfane sulfur has not been sufficiently studied; however, it is obvious that it exists at high contents in cells/tissues. Numerous publications indicate the high antioxidant activity of (hydro)polysulfides [14,53,54]. For example, the specific ability of GS_3_G to neutralize H_2_O_2_ in the presence of glutathione reductase was demonstrated in a model system [18]; diallyl trisulfide suppressed the H_2_O_2_-induced activation of hepatic stellate cells and attenuated the effects of oxidative stress and lipid metabolism impairments in rat models of metabolic syndrome and cardiac ischemia–reperfusion [55,56]. However, at the same time, it was revealed that CysS_3_Cys in high concentrations is capable of inhibiting cellular metabolism and proliferation and quickly inducing cellular and ER stress, accompanied by widespread protein-thiol oxidation [57].

Using a culture of THP-1 monocytic cells, we investigated whether the ALSS of cellular proteins changes in vitro under chronic oxidative and reductive stress. Oxidative stress, a condition in which reactive oxygen species are generated beyond the cell’s compensatory capacity, was induced by adding sodium arsenide to the incubation medium. A model system containing chloramine T (a source of nitrogen-centered radicals), but not metal-catalyzed oxidation (ascorbic acid + Fe^3+^), showed that it caused a time-dependent decrease in the content of sulfane sulfur of HSA [33]. The literature contains numerous studies demonstrating the ability of H_2_S to attenuate oxidative stress, but we found no data on the effect of arsenide-induced oxidative stress on H_2_S or polysulfide production by cells, and the results of studies using H_2_O_2_-induced stress are contradictory. For example, the exposure of vascular smooth muscle cells (VSMCs) to H_2_O_2_ increased H_2_S production due to CSE activation [58]. However, H_2_O_2_ caused a decrease in H_2_S production in mitochondria isolated from murine hepatoma cells due to the inhibition of MST activity [59].

Radical forms of ROS (O_2_·^−^) and peroxides are capable of breaking polysulfide chains and converting them into alkyl polysulfide radicals (RS_n_S·), the recombination of which restores the original structure or leads to oxidation to sulfoxides or sulfones [60]. However, reactions transferring sulfonic sulfur from polysulfides to low-molecular-weight thiols are believed to be of greater physiological significance. This results in the formation of supersulfides, such as GSS_n_H, which are more effective at neutralizing ROS than thiols or H_2_S [18]. These reactions are likely responsible for the depletion of the ALSS pool under OS conditions.

Reductive stress, a condition in which reactive oxygen species levels are excessively suppressed, was modeled by adding dithiothreitol to the culture medium. This dithiol scavenges reactive oxygen species and reduces thiols oxidized to disulfides and hydropolysulfides. Dithiothreitol itself does not affect the content of sulfane sulfur, as shown in a model system with albumin [26]. Under reductive stress, protein disulfide bonds form inappropriately, resulting in the activation of the unfolded protein response [61], and H_2_S production is also activated [62,63]. Our results show that despite the decrease in protein content in cells, the amount of alkali-labile sulfonic sulfur in proteins increases under conditions of chronic reductive stress, which may be directly related to the synthesis of H_2_S.

We also investigated the effect of hyperhomocysteinemia, a condition characterized by elevated Hcy levels, an intermediate metabolite of methionine, on plasma ALSS. Chronic hyperhomocysteinemia is typically modeled by increasing methionine levels in animal diets [64,65]. This condition is also associated with oxidative stress and pathological processes (endothelial dysfunction, atherosclerosis, and hypertension) and is considered a risk factor for cardiovascular and neurodegenerative diseases [66]. Upon entering cells, methionine is converted to S-adenosylmethionine, which is an allosteric activator of CBS that also stabilizes the enzyme against proteolytic degradation [67]. This, in turn, should accelerate the conversion of Hcy to Cys and increase the latter’s level, which is consistent with our results. Thus, on the one hand, a methionine diet is accompanied by an increase in not only sulfur consumption but also by the content of H_2_S synthesis substrates and sulfane sulfur transporters. On the other hand, the activity of CBS and CSE is regulated, in different directions, by the redox balance of pairs of Cys residues (Cys-272 and Cys-275 for CBS and Cys252 and Cys255 for CSE) [58,62], which can also undergo changes in hyperhomocysteinemia. It was found that under conditions of chronic hyperhomocysteinemia, a decrease in endogenous H_2_S production is observed due to a decrease in the expression and activity of CBS, CSE, and MST [68,69]. Moreover, despite the fact that excess homocysteine is also accompanied by an increase in the level of the transmethylation inhibitor S-adenosylhomocysteine, it was found that CSE transcription is suppressed, on the contrary, by the hypermethylation of its promoter [70]. Our results show that hyperhomocysteinemia is accompanied by a decrease in plasma ALSS, which indicates the important role of H_2_S in maintaining the pool of protein-bound polysulfides.

Stroke is one of the most socially significant pathologies. Elevated homocysteine levels are often observed in patients and are a risk factor for stroke. Lowering these levels also helps reduce the risk of stroke [71]. In our study, ALSS in the plasma of patients with stroke was lower than in control samples, and homocysteine levels, on the contrary, were higher. This is generally consistent with the results obtained with the rat hyperhomocysteinemia model, suggesting an important role for homocysteine as a marker or factor influencing the polysulfide pool. However, in addition to homocysteine, many other factors can significantly influence ALSS levels. For example, acute cerebral ischemia is known to be accompanied by a systemic stress response, leading to a massive release of reactive oxygen species in peripheral vessels [72]. The content of thiol groups in blood plasma is significantly reduced both in experimental models and clinical observation [36,73]. Therefore, reduced ALSS levels may be a consequence of both high homocysteine and this systemic metabolic stress response to stroke. Clearly, further research is needed here.

## 5. Conclusions

It is becoming increasingly clear that the alkaline-labile sulfonic sulfur pool is not an artifact, but a large, yet underestimated, depot of H_2_S, and it therefore plays an important role in physiology and pathology, especially in antioxidant defense mechanisms.

This study presents an improved method for the evaluation of ALSS in various biological matrices (plasma and blood cells, internal organs, and cell cultures). The specificity of this method is approximately 80%, which limits its applicability as a quantitative method for determining (hydro)polysulfides. However, the simplicity of the approach and the availability of reagents make it a suitable tool for large-scale clinical trials. We found that the generation of H_2_S from polysulfides does not require the presence of reducing agents and that ALSS in cells and blood plasma decreases under conditions of oxidative stress and hyperhomocysteinemia, respectively. Therefore, this indicator holds promise as a new biomarker for sulfur metabolism disorders associated with oxidative stress.

## Figures and Tables

**Table 1:** ALSS of low-molecular-weight thiols (*n* = 4).

Thiol	H_2_S/Thiol
Cys	<0.001
N-Ac-Cys	<0.001
Na_2_S_2_O_3_	0.0014 ± 0.0008
Methionine	0.0023 ± 0.0016
Cystine	0.0151 ± 0.0008
Cysteinylglycine	0.0235 ± 0.0025
GSH	0.065 ± 0.008
GSSG	0.199 ± 0.0125

**Table 2:** ALSS evaluation of commercial protein preparations (*n* = 4).

Protein	Origin	ALSS, μmol/g	H_2_S/Protein	Cys/Protein	H_2_S/Cys
BSA 96%	Bovine	268 ± 10	18.5 ± 0.7	35	0.528
BA	Bovine	258 ± 2	17.8 ± 0.14	35	0.509
HSA 95%	Human	274 ± 10	18.4 ± 0.64	35	0.526
Collagen	Bovine	4.56 ± 1.08	1.37 ± 0.32	-	-
Collagen I type	Rat	4.94 ± 0.81	1.48 ± 0.24	-	-
Protamine	Clupea pallasii	1.81 ± 0.31	0.006 ± 0.001	-	-
Hb	Human	13.4 ± 0.3	0.87 ± 0.02	6	0.145
Insulin 100 ME/mL	Human	493 ± 18	2.86 ± 0.1	6	0.476
Trypsin	Bovine	178.9 ± 8.2	4.3 ± 0.2	12	0.358
Fibrinogen	Bovine	65.2 ± 3.2	22.2 ± 1.1	58	0.383

**Table 3:** ALSS of human and rat blood plasma, showing the effect of a methionine diet on ALSS.

Group (N)	ALSS, mM	Total Plasma Protein, g/L	ALSS, μmol/g Protein
Control humans (10)	10.9 (8.45; 11.5)	56.5 (53.3; 64.6)	177.6 (171.8; 183.6)
Stroke patients (20)	9.34 (8.61; 10.43)	62.1 (57.4; 72.2)	145.0 ^£^ (133.5; 149.2)
Control rats (15)	9.03 (8.69; 9.44)	70.7 (66.3; 72.4)	133.3 * (127.6; 135.2)
Methionine diet rats (14)	8.43 (7.49; 8.95)	68.6 (65.3; 75.6)	122.8 ** (115.8; 130.5)

* Corrected *p* = 6 × 10^−7^ (control humans vs. control rats). ** corrected *p* = 0.036 (control rats vs. methionine diet). ^£^ corrected *p* = 4 × 10^−5^ (control humans vs. stroke patients).

**Table 4:** ALSS evaluation in internal organs of rats (*n* = 8).

Organ	ALSS, μmol/g Tissue	ALSS, μmol/g Protein
Liver	15.78 (14.68; 16.54) min—10.33, max—21.06	59.8 (55.6; 60.7) min—45.3, max—79.0
Brain	8.25 (7.31; 8.38) min—6.50, max—8.67	48.1 (42.1; 50.6) min—37.7, max—57.8
Heart	11.56 (9.38; 12.60) min—8.50, max—14.56	45.6 (39.2; 55.9) min—29.5, max—69.2

## Data Availability

The data presented in this study are available upon request from the corresponding author due to protecting the privacy of the participants.

## Supplementary Materials

The following supporting information can be downloaded at https://www.mdpi.com/article/10.3390/biom16060784/s1, Figure S1: Effect of BSA amount in sample on ALSS evaluation; 50 μL of BSA (2.5–50 g/L) in PBS (*n* = 4) was prepared as described in Section 2.6; Figure S2: Influence of incubation time on H_2_S generation from BSA (10 g/L) at 95 °C. Samples (*n* = 4) were prepared as described in Section 2.6. * *p* = 2.2 × 10^−5^; Figure S3: Comparison of Na_3_PO_4_ and K_3_PO_4_ as incubation buffers to assess the ALSS of a model BSA mixture (10 g/L in PBS). Samples (*n* = 4) were prepared as described in Section 2.6, but the SDS solution was replaced with water; Figure S4: Optimization of the composition of the model mixture for MB synthesis: (A) 20 mg/L Na_2_SxH_2_O, 8.7 mM DMPD, 13 mM FeCl_3_, and 0.25–1.5 M HCl, 10-fold dilution prior photometry; (B) 5 mg/L Na_2_SxH_2_O, 8.7 mM DMPD, 0.8 M HCl, and 4–24 mM FeCl_3_, 3-fold dilution prior photometry; (C) 10 mg/L Na_2_SxH_2_O, 8 mM FeCl_3_, 0.8 M HCl, and 2–24 mM DMPD, 10-fold dilution prior photometry (*n* = 3); Figure S5: Absorption spectrum of the reaction mixture (14 mM DMPD, 8 mM FeCl_3_ in 0.8 M HCl) after 30 min incubation and two-fold dilution with water; Figure S6: Scheme of polysulfide degradation using TCEP and sulfite; Figure S7: (A) Stability of model mixture extracts (100 mg/L Na_2_SxH_2_O) during storage (25 °C, dark). (B) Effect of plasma storage time (37 °C, dark) on ALSS. (C) Effect of repeated freeze-thawing on plasma ALSS. All samples were prepared as described in Section 2.6 (*n* = 4); Figure S8: Effect of additives on ALSS BSA (50 μL, 10 g/L). The protein sample was supplemented with 50 μL of 5 mM Cys/cystine (in 0.1 M HCl) or 2.7% H_2_O_2_ (*n* = 4). Samples were processed and analyzed as described in Section 2.6. Samples containing cystine were additionally neutralized with 50 μL of 0.1 M NaOH; Table S1: Completeness of H_2_S precipitation by zinc acetate on a model mixture (*n* = 3).

## Abbreviations

The following abbreviations are used in this manuscript:

ACN	acetonitrile
ALSS	alkalinelabile sulfane sulfur
BA	bovine albumin, heat shock isolation
BC	blood cells
BSA	bovine serum albumin
CBS	cystathionine-β-synthase
CE	capillary electrophoresis
CSE	γ-cystathionase
Cys	cysteine
DMPD	N,N-Dimethyl-p-phenylenediamine
DMSO	dimethyl sulfoxide
DTT	dithitreitol
EMSP	Elimination Method of Sulfide from Polysulfide
ETHE1	ethylmalonic encephalopathy protein 1
ER	endoplasmic reticulum
FA	formic acid
GADPH	Glyceraldehyde 3-phosphate dehydrogenase
GC	gas chromatography
GSH	glutathione
GSSG	glutathione disulfide
Hb	hemoglobin
Hcy	homocysteine
HEPES	4-(2-hydroxyethyl)-1-piperazineethanesulfonic acid
HPLC	high-performance liquid chromatography
HSA	human serum albumin
LPE	liquid-phase extraction
MB	methylene blue
MS	mass spectrometry
MST	3-mercaptopyruvate sulfurtransferase
NMDA	N-methyl-D-aspartate
PBS	phosphate-buffered saline
ROS	reactive oxygen species
RSS	reactive sulfur species
SDS	dodecyl sulfate Na
SOD	superoxide dismutase
TRPA1	Transient Receptor Potential Ankyrin 1
