# Supplementary material for: Evaluation of Alkaline-Labile Sulfane Sulfur in Biological Samples: The Influence of Hyperhomocysteinemia and Oxidative Stress
Source: Biomolecules. 2026 May 27;16(6):784. doi: 10.3390/biom16060784 (PMC13296428; doi:10.3390/biom16060784)
Supplement: Supplementary file 1 [file biomolecules-16-00784-s001.zip › biomolecules-4292981-supplementary.pdf]

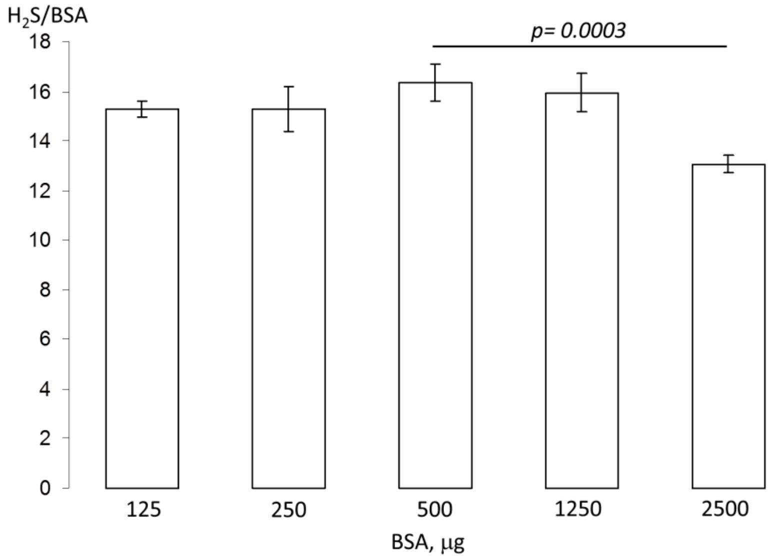

Figure S1. Effect of BSA amount in sample on ALSS evaluation; 50 µl of BSA (2.5-50 g/L) in PBS (*n* = 4) was prepared as described in Section 2.6.

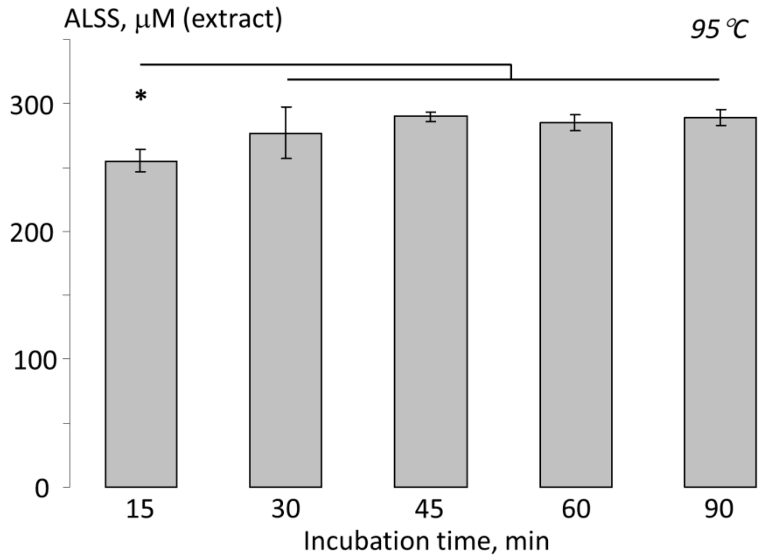

Figure S2. Influence of incubation time on H_2S generation from BSA (10 g/L) at 95°C. Samples (*n* = 4) were prepared as described in Section 2.6. * $p = 2.2 \cdot 10^{-5}$.

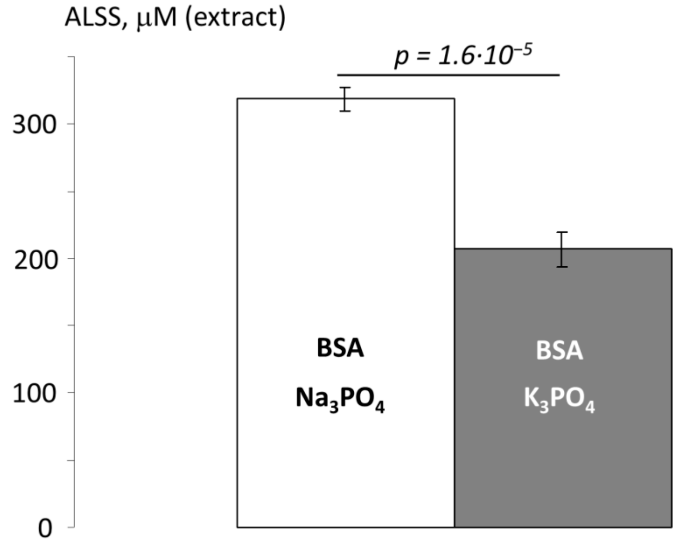

Figure S3. Comparison of Na_3PO_4 and K_3PO_4 as incubation buffers to assess the ALSS of a model BSA mixture (10 g/L in PBS). Samples (n = 4) were prepared as described in Section 2.6, but the SDS solution was replaced with water.

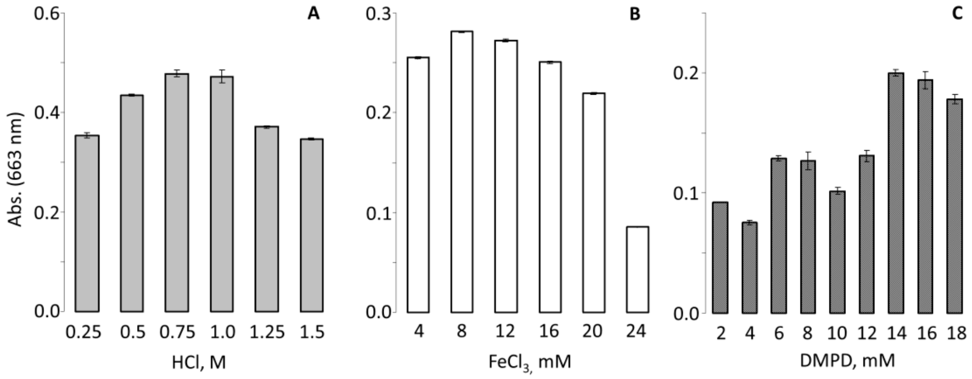

Figure S4. Optimization of the composition of the model mixture for MB synthesis: A—20 mg/L Na_2SxH_2O, 8.7 mM DMPD, 13 mM $FeCl_3$, and 0.25-1.5 M HCl, 10-fold dilution prior photometry; B—5 mg/L Na_2SxH_2O, 8.7 mM DMPD, 0.8 M HCl, and 4-24 mM $FeCl_3$, 3-fold dilution prior photometry; C—10 mg/L Na_2SxH_2O, 8 mM $FeCl_3$, 0.8 M HCl, and 2-24 mM DMPD, 10-fold dilution prior photometry (n = 3).

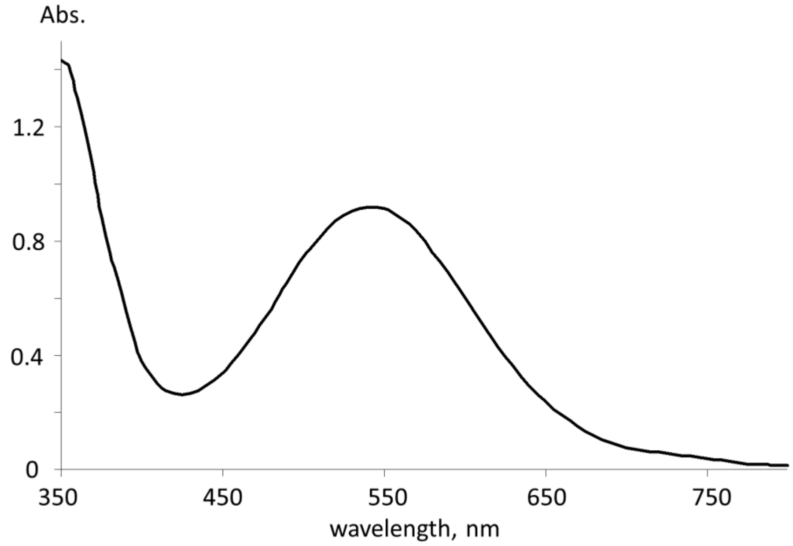

Figure S5. Absorption spectrum of the reaction mixture (14 mM DMPD, 8 mM $FeCl_3$ in 0.8 M HCl) after 30-minute incubation and two-fold dilution with water.

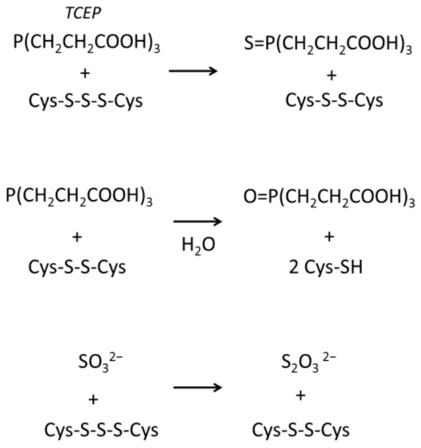

Figure S6. Scheme of polysulfide degradation using TCEP and sulfite.

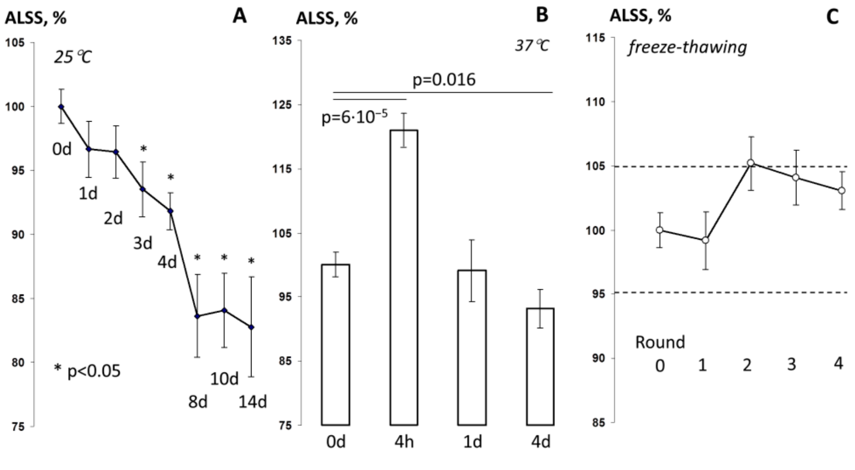

Figure S7. A—Stability of model mixture extracts (100 mg/L Na_2SxH_2O) during storage (25°C, dark). B—Effect of plasma storage time (37°C, dark) on ALSS. C—Effect of repeated freeze-thawing on plasma ALSS. All samples were prepared as described in Section 2.6 (n = 4).

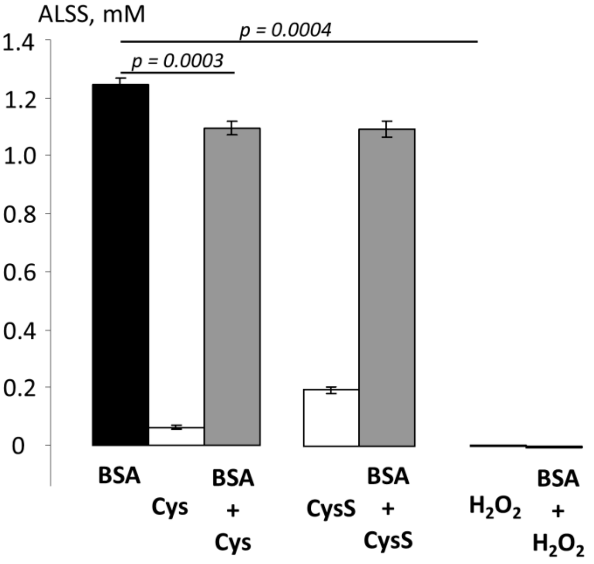

Figure S8. Effect of additives on ALSS BSA (50 µL, 10 g/L). The protein sample was supplemented with 50 µL of 5 mM Cys/cystine (in 0.1 M HCl) or 2.7% H_2O_2 (n = 4). Samples were processed and analyzed as described in Section 2.6. Samples containing cystine were additionally neutralized with 50 µL of 0.1 M NaOH.

Table S1. Completeness of H_2S precipitation by zinc acetate on a model mixture (n = 3)

Series	H_2S in the supernatant, µM	H_2S recovery, %
blank	0.035±0.002	0±4.8
Na_2S+$ZnAc_2$	4.318±0.120	97.7±2.8 (pellet)
Na_2S	187.3±6.1	100±3.3 (supernatant)
